# Immediate post-operative PDE5i therapy improves early erectile function outcomes after robot assisted radical prostatectomy (RARP)

**DOI:** 10.1007/s11701-021-01198-4

**Published:** 2021-02-08

**Authors:** Arjun Nathan, Shivani Shukla, Amil Sinha, Sailantra Sivathasan, Amir Rashid, Joseph Rassam, Sonny Smart, Keval Patel, Nimish Shah, Benjamin W. Lamb

**Affiliations:** 1 Urology Department, Addenbrooke’s Hospital, Cambridge, UK; 2 University of Cambridge, Cambridge, UK; 3 University College London, London, UK

**Keywords:** Continence, Erectile dysfunction, Phosphodiesterase inhibitors (PDE5i), Robot-assisted radical prostatectomy (RARP)

## Abstract

To assess whether the timing of post-operative Phosphodiesterase Inhibitor (PDE5i) therapy after Robot-Assisted Radical Prostatectomy (RARP) is associated with a change in early erectile function (EF) outcomes, continence or safety outcomes. Data were prospectively collected from a single surgeon in one tertiary centre. 158 patients were treated with PDE5i therapy post RARP over a 2-year period. PDE5i therapy was started: immediately (day 1–2) post-op in 29%, early (day 3–14) post-op in 37% and late (after day 14) post-op in 34%. EPIC-26 EF scores were collected pre-op and post-op. There were no significant differences in pre-operative characteristics between the therapy groups. Drop in EF scores and percentage return to baseline for unilateral nerve sparing was, respectively, 9 and 11.1% of immediate therapy, 7 and 14.8% of early therapy and 9.7 and 9.5% of late therapy (*p* = 0.9 and *p* = 0.6). For bilateral nerve sparing, this was, respectively, 3.5 and 42.9% immediate therapy, 5.5 and 35.5% early therapy and 7.3 and 25% late therapy (*p* = 0.017 and *p* = 0.045). Pad free and social continence were achieved in 54% and 37% of those receiving immediate therapy, 60% and 33% for early therapy and 26% and 54% for late therapy. There were no differences in compliance, complication or readmission outcomes. In patients with bilateral nerve sparing RARP, immediate post-operative PDE5i therapy can protect EF and improve early continence outcomes. Therefore, immediate PDE5i therapy should be considered in patients following nerve sparing RARP to maximise functional outcomes.

## Introduction

Robot-Assisted Radical Prostatectomy (RARP) is a minimally invasive procedure which has been rapidly adopted by urological surgeons worldwide. Despite the learning curve and costs involved, RARP has shown equal or better cancer and quality outcomes compared to open surgery [1].

Despite the benefits of RARP, post-operative Erectile Dysfunction (ED) continues to be an issue for patients. A meta-analysis of potency rates by Ficcara et al*.* [2] reported the incidence of ED in men undergoing either unilateral or bilateral nerve sparing to be 68%, 47%, 31% and 37% at 3-, 6-, 12- and 24-month follow-up intervals. When considering bilateral nerve-sparing, only the ED rates were better at 44%, 31%, 26%, and 18%, respectively. Return to baseline function takes approximately six months to two years [3]. Furthermore, early potency rates post RARP can be as low as 33% during the 6-month follow-up period [4]. Salvage RARP has shown inferior Erectile Function (EF] outcomes compared to primary RARP with only 5% maintaining their pre-operative EF with two-year follow-up [5].

Crucially, EF has been shown to have significant effects on the psychological wellbeing of patients with studies showing over half of patients (51.6%] can feel angry, bitter or depressed at the loss of potency [6].

As well as being affected by age, BMI, co-morbidities, baseline erectile function and nerve-sparing, EF outcomes are also affected by the use of penile rehabilitation therapy following surgery [7].

The use of phosphodiesterase inhibitors (PDE5i) following RARP, as part of penile rehabilitation, has been shown to preserve EF. ED post RARP can be explained by intra-operative damage to the pelvic splanchnic nerves. Upon sexual stimulation, the pelvic splanchnic nerves release Nitric Oxide (NO) from Non-Noradrenergic Non-Cholinergic (NANC) fibres and Acetylcholine (ACh) from parasympathetic fibres leading to an overall increase in cyclic-GMP (cGMP) concentration. Subsequently there is a reduction in calcium ion concentration leading to the relaxation of vascular smooth muscle. Laboratory studies have demonstrated that PDE5i’s block the action of phosphodiesterase which acts to break down cGMP, thereby working to reduce calcium ion concentration and promoting relaxation of vascular smooth muscle [8].

Furthermore, numerous clinical studies have shown that the use of PDE5i’s following RARP improve EF for patients with nerve-sparing surgery. Briganti et al*.* followed patients treated by high-volume surgeons with bilateral nerve sparing over a period of three years. They found EF recovery rates to be significantly higher (73%) in the PDE5i therapy group compared to the placebo (37%) group [9]. Similarly, Nelson et al*.* found the EF recovery rate in the PDE5i group to be 43% compared to the placebo group of 22% at 2-year post-op [10]. Mulhall et al*.* found that three months of on-demand 200 mg Avanafil treatment significantly improved EF, with outcomes of 41% in the treatment group and 10.7% in the placebo group [11]. Current favourable evidence is better established for bilateral nerve sparing compared to unilateral nerve sparing [12]. The limited evidence for PDE5i therapy for patients with no nerve sparing shows no benefit [13].

Despite considerable evidence supporting the efficacy of PDE5i therapy on EF post RARP, there are few guidelines or evidence-based recommendations advising the optimal time to begin PDE5i therapy to begin post-RARP. Teloken et al*.* questioned 301 doctors from 41 countries and found 95% use PDE5i therapy post RARP; however, only 6% initiate therapy immediately post-operatively. 54% waited until after the catheter removal around two weeks and the remaining 40% all started therapy at some point in the first 4 months [14]. Moreover, some studies have suggested that the early use of PDE5i may be associated with an increased incidence of adverse events and worsen early continence outcomes [15, 16].

## Aim

We aim to assess if there is an association between the timing of initiation of PDE5i therapy and early EF outcomes. Additionally, we aim to assess if the timing of initiation of PDE5i therapy is associated with adverse events or if it affects continence outcomes.

## Subjects/patients and methods

Data were prospectively collected from a high-volume, single surgeon at a tertiary centre. Data were then retrospectively evaluated. All patients were registered as part of The BAUS national outcomes audit and registered with the institutional audit department (Ref. PRN8750). 187 patients who underwent primary RARP were identified over a 2-year period from 2018 to 2020, from these 158 patients were treated with PDE5i therapy. 29 patients did not receive PDE5i therapy due to non-nerve sparing status or lack of patient interest and were excluded. 54, 58 and 46 patients were started on PDE5i therapy late (> 14 days), early (3–14 days) and immediately (1–2 days) post-operatively.

The decision to attempt nerve sparing surgery was made jointly between the operating surgeon and patient based on a synthesis of patient priorities and disease factors. Specifically, patient priorities included the relative prioritisation of functional recovery and oncological control. Disease factors included the location lesion on the MRI scan, PSA level, as well as the distribution and grade of biopsy cores with PCa. Finally, digital rectal examination under anaesthesia was performed at the start of the case. The definition of nerve sparing included intrafascial and interfascial, as well as high- and low-release of the neurovascular bundle. The execution of nerve sparing reflected the intent as set out above, as well as what was intraoperatively feasible. All patients gave informed consent for surgery and PDE5i therapy. All patients were included in the analysis regardless of baseline EF or nerve-sparing status. All patients were seen pre-operatively by a physiotherapist to learn Pelvic Floor Muscle Training (PFMT) before surgery and at TWOC and were instructed to perform PFMT manoeuvres three times per day [17].

Three cohorts were stratified based on the time PDE5i therapy was initiated post RARP. Tadalafil 5 mg once daily was the PDE5i of choice. In 2018, patients were started on late therapy (> 14 days) as was standard and existing practice. However due to emerging evidence suggesting, early PDE5i therapy may improve penile rehabilitation, practice was changed to start therapy immediately post-op (1–2 days) [18, 19]. Later, due to concerns regarding safety and possible increased bleeding risk, initiation time was delayed to early post-operative therapy (3–14 days) [15]. Some patients received specific treatment depending on individual risk factors, such as nerve-sparing status and risk of haematoma. This was not an experimental study rather an evaluation of clinical practice based on emerging evidence to improve patient care and outcomes. All patients had existing co-morbidities taken into account and were counselled appropriately regarding contraindications of PDE5i therapy.

EF and post-operative continence were measured using the patient reported outcome measure (PROM)—The Expanded Prostate Cancer Index Composite Short Form (EPIC-26). EPIC-26 is a validated tool for comprehensive assessment of men with PCa. EF was measured using questions 8–11 with a minimum score of 5/24 and a maximum score of 24/24 [20].

Pre-operative metrics, such as age, BMI, Charlson Co-Morbidities and intra-operative nerve spare status, as well as post-operative data on continence, complications, readmission, adherence and discontinuation, were also collected.

Return to Baseline was defined as the post-operative EF score equalling or exceeding the pre-operative EF score. Full continence was defined as using zero pads per day, social continence was defined as using one pad per day and incontinence was defined as using two or more pads per day [21, 22].

Data collection, tables and figures were completed using Microsoft Excel 2019. Statistical analysis was done using SPSS 26th Edition, IBM. Chi-Squared tests were used to compare categorical data and independent *T* tests and ANOVA were used to compare continuous data.

## Results

### Demographics

There were no statistically significant differences in age, BMI, Co-Morbidities or pre-operative EF scores between the three groups. 53% of our cohort had bilateral nerve-sparing, 42% had unilateral nerve-sparing and 6% had no nerve-sparing (Table 1). The median follow-up time was 43 days (IQR: 41–47) and there was no statistical difference between the groups.

**Table 1:** Pre, intra and post-operative characteristics of therapy groups by PDE5i therapy onset

Criteria (day post-op)	Total	Immediate	Early	Late	*P* value
		1–2	3–14	> 14	
*N* (%)	158 (100)	46 (29)	58 (37)	54 (34)	
Pre-operative					
Median age (IQR) years	64 (59–67)	66 (62–68)	62 (57–66)	64 (59–67)	0.916
Median BMI (IQR)	27 (25–30)	28 (25–30)	27 (25–30)	27 (24–29)	0.854
Median ASA	2	2	2	2	0.935
CCI					
0	138	40	50	48	0.711
1	13	4	6	3	
2	5	2	1	2	
3	2	0	1	1	
CCI (age-adjusted)					
0–1	138	40	51	47	0.639
2	4	1	1	2	
> = 3	16	5	6	5	
Intra-operative					
Nerve-sparing status					
Nil *N* (%)	9 (6)	0 (0)	0 (0)	9 (6)	0.210
Unilateral *N* (%)	66 (42)	18 (11)	27 (17)	21 (13)	
Bilateral *N* (%)	83 (53)	28 (18)	31 (20)	24 (15)	
Post-operative					
Erectile function					
Mean pre-Op EFs	17.4	17.9	18.2	16.2	0.110
Mean drop EF	7	5.7	6.6	8.6	0.097
Return to baseline *N* (%)	39 (25)	14 (36)	15 (39)	10 (26)	0.374
Continence					
Full continence *N* (%)	74 (47)	25 (54)	35 (60)	14 (26)	0.001*
Social continence *N* (%)	65 (41)	17 (37)	19 (33)	29 (54)	0.043*
Incontinence *N* (%)	19 (12)	4 (9)	4 (7)	11 (20)	0.065
Safety					
Stopped *N* (%)	21 (13)	6 (13)	3 (5)	12 (22)	0.589
Due to side effects *N* (%)	11 (7)	5 (11)	2 (3)	4 (7)	0.934
Due to compliance (%)	10 (6)	1 (2)	1 (2)	8 (15)	0.574
Complications *N* (%)	8 (5)	2 (4)	2 (3)	4 (7)	0.948
Readmissions *N* (%)	3 (2)	1 (2)	1 (2)	1 (2)	0.865

*N* Numbers, *CCI* Charlson Comorbidity Index, *EFs* Erectile Function Score

*Statistically significant at *p* =  < 0.05

Full continence = 0 pads, Social continence = 1 pad/day, Incontinence = 2 or more pads/day

### Erectile function outcomes

There was a mean drop in EF score of 9.4 and return to baseline of 22% for no nerve sparing. Unilateral nerve sparing achieved 8.8 and 12% whilst bilateral achieved 5.4 and 35% (*p* < 0.05) (Table 2).

**Table 2:** Erectile Function Outcomes by nerve-sparing groups and PDE5i therapy onset

Nerve spare				
	Nil	Unilateral	Bilateral	*P* Value
*N* (%)	9 (6)	66 (42)	83 (53)	
Mean drop EFs	9.4	8.8	5.4	0.005*
Return to baseline (%)	2 (22)	8 (12)	29 (35)	0.005*

Nerve spare and PDE5i Onset												
Nerve-sparing	Total	Immediate				Early				Late		
		Nil	Uni		Bi	Nil	Uni	Bi		Nil	Uni	Bi
*N*	158	0	18		28	0	27	31		9	21	24
Mean pre-Op EFs	17.4	–	18.2		17.7	–	17	19.2		14.7	16.1	16.9
Mean drop EFs	7	–	9		3.5	–	7	5.5		9.4	9.7	7.3
Return to baseline (%)	39 (24.7)	–	2 (11.1)		12 (42.9)	–	4 (14.8)	11 (35.5)		2 (22.2)	2 (9.5)	6 (25.0)
Statistical comparison												
Nerve-Sparing				Unilateral (*p* value)					Bilateral (*p* value)			
EF score change												
Immediate vs. late				+ 0.6 (1.0)					+ 3.8 (0.04)*			
Early vs. late				+ 1.6 (0.9)					+ 1.8 (0.9)			
Immediate vs. early				+ 1 (0.9)					+ 1.9 (0.9)			
Return to Baseline %												
Immediate vs. late				+ 1.6 (0.6)					+ 17.9 (0.05)*			
Early vs. late				+ 5.3 (0.5)					+ 10.5 (0.05)*			
Immediate vs. early				− 3.7 (0.8)					+ 7.4 (0.1)			

*N* Numbers, *Uni* Unilateral, *Bi* Bilateral, *EFs* Erectile Function Score

*Statistically significant at *p* =  < 0.05

The mean pre-operative EF score was 17.4 out of 24, mean drop in EF score was 7 and the return to baseline was 25%. Mean drop in EF and return to baseline was 5.7 and 36% with immediate therapy, 6.6 and 39% with early therapy and 8.6 and 26% with late therapy (*p* = 0.097 and *p* = 0.374) (Table 1).

For unilateral nerve spare, mean drop in EF score and return to baseline was 9 and 11% for immediate, 7 and 15% for early and 9.7 and 10% for late. For bilateral nerve spare, mean drop in EF score and return to baseline was 3.5 and 43% for immediate, 5.5 and 36% for early and 7.3 and 25% for late (*p* < 0.05) (Table 2).

11 patients were less than 55 years, 75 patients were between 55 and 64 and 72 patients were above 64. Return to baseline was achieved in 36%, 20% and 28%, respectively (*p* = 0.356). Mean drop in IIEF was 5.5, 7.6 and 6.7, respectively (*p* = 0.555).

### Continence outcomes

Overall, in our entire cohort, full continence was achieved by 47%, social continence 41%, with 12% remaining incontinent, at a follow-up of 6–8 weeks. Between therapy groups, rates of full and social continence were 54% and 37% in immediate therapy, 60% and 33% in early therapy and 26% and 54% in late therapy (*p* < 0.05) (Table 1).

### Safety outcomes

PDE5i therapy, in the total cohort, was stopped in 13%, with 7% stopping due to side effects and 6% due to non-compliance. Side effects included headache (*n* = 6), dizziness (*n* = 3) and gastro-intestinal issues (*n* = 2). From the entire cohort, 2% were readmitted and 5% developed 90-day complications of which one was a haematoma, managed conservatively. There were no statistically significant differences between the three groups for discontinuation, complications or readmission (Table. 1).

## Discussion

Our results suggest that patients who were started earlier on PDE5i therapy after RARP enjoyed better EF outcomes overall. Specifically, the mean drop in EF score showed a trend towards being reduced in the immediate (5.7) and early (6.6) therapy groups compared to late therapy (8.6) group for all nerve sparing groups, though this did not reach statistical significance (*p* = 0.097). Furthermore, return to baseline EF score trended towards improvement in the immediate (36%) and early (39%) groups compared to 26% in late group, though this was not statistically significant (*p* = 0.374).

However, when utilising nerve sparing techniques, immediate therapy showed significant benefit compared to late therapy. These results were more pronounced in bilateral nerve sparing. For bilateral neve sparing, immediate therapy had a higher EF score by 3.8 compared to late therapy (*p* = 0.04). Additionally, return to baseline in immediate therapy was 17.9% higher compared to late therapy (*p* = 0.05) and early therapy was 10.5% higher than late therapy (*p* = 0.05). Immediate and early PDE5i therapy in unilateral nerve sparing also showed greater EF outcomes compared to late therapy; however, the differences were not statistically significant. Further, in younger patients, return to baseline was greater and mean drop in EF score was less. This was not statistically significant but suggests that younger age is a favourable factor in EF recovery.

Continence outcomes were also better in immediate or early therapy compared to late therapy for all patients. Full continence and social continence were achieved by 54% and 37% in the immediate group, 60% and 33% in the early group and only 26% and 54% in the late group (*p* = 0.001). Additionally, there were no differences in compliance, complications or readmissions between the three therapy groups.

A novel finding from our data showed 11% (*n* = 18) of our cohort recovered EF above baseline. This is possibly due to untreated pre-existing erectile dysfunction. In this cohort, median pre-operative EF was 11 with a median increase of 4.

Our results show that the level of preservation of the neurovascular bundle is important in erectile function recovery post RARP, as previously described [2]. For example, Greco et al*.* [23] showed 69% return to sexual intercourse with bilateral nerve sparing compared to 43% in unilateral. Additionally, Sridhar et al*.* [13] found that for patients with non-nerve sparing surgery, post-operative treatment with PDE5i made no significant difference to EF.

PDE5i therapy is a well-established component of penile rehabilitation after RARP. Montorsi et al*.* [18] showed significantly greater EF scores using on-demand Sildenafil compared to placebo. Pace et al*.* [24] also showed improved potency rates using Sildenafil (87%) compared to placebo (56%). Generally, starting PDE5i therapy earlier post-operatively showed better EF score results [25]. However, our study adds further weight to the previous limited evidence that earlier post-operative PDE5i therapy improves EF. We showed a mean drop in EF and return to baseline was 5.7 and 36% in the immediate group, 6.6 and 39% in the early group and 8.6 and 26% in the late group. Comparison of different PDE5i has shown 71% of patients preferred Tadalafil and 29% preferred Sildenafil due to side effect and erection profile [26].

Studies have shown that PDE5i therapy after RARP may also improve urinary continence outcomes [27]. Kaiho et al*.* [16] showed that immediate PDE5i therapy may temporarily worsen immediate incontinence compared to late therapy. However, our data showed an improvement in early continence rates with immediate PDE5i therapy compared to late therapy. Furthermore, considering anecdotal and pharmacological concerns regarding increased bleeding, our study did not show any differences in adverse effects when initiating PDE5i therapy immediately after RARP [28].

Numerous studies have documented the poor quality of life associated with ED, with most suffering from moderate to severe psychological effects. Common concerns reported included partnership issues, guilt, anger and self-deprecation [29].

Rates of ED after RARP have been reported to be as high as 68% and non-surgical interventions, such as focal therapy and radiotherapy, report lower rates of ED [2, 30]. Therefore, immediate PDE5i therapy after RARP should be considered to achieve the best possible EF outcome for patients and improve overall satisfaction after RARP.

Despite multiple studies proving the benefit of PDE5i therapy after RARP, there is no clear consensus or pathway implemented for the management of ED after RARP. In general, PDE5i therapy is started four to six weeks post-operatively; however, our results show that immediate or early PDE5i therapy can improve EF outcomes with no adverse effects. We recommend the use of immediate PDE5i therapy for patients undergoing nerve sparing RARP to improve EF outcomes, continence outcomes and patient satisfaction.

Limitations of this study include single-surgeon data with an associated surgical learning curve, however, prior to the start of the study period the primary surgeon had carried out over 100 RARPs and participated in over 200 more during training. EPIC26, a validated tool for assessment of men with PCa, was used instead of the International Index of Erectile Function (IIEF) at this centre. The study lacks randomisation and data are only assessed at a short follow-up interval. However, our study shows that immediate PDE5i therapy is safe and therefore further studies with longer-term follow-up are warranted.

Further research with randomisation of patients in multiple centres with longer follow-up would improve the quality of the results. Pre-operative PDE5i therapy may also show a benefit to EF outcomes. Additionally, a cost–benefit analysis of extended PDE5i use with respect to quality of life should be undertaken.

## Conclusion

In conclusion, immediate or early post-operative PDE5i therapy after RARP trends towards better early EF outcomes compared to late therapy. This effect is significantly more pronounced in patients undergoing bilateral nerve sparing. There may also be a benefit in EF for patients with unilateral nerve sparing; however, the effects are less pronounced. Immediate therapy can also improve early continence outcomes and does not show an increase in adverse outcomes. Preservation of sexual function and regaining continence are important factors for patients undergoing RARP and therefore immediate therapy should be considered especially in bilateral nerve sparing cases.

## Data Availability

SPSS code for statistical analysis is not available.

## Availability of data

Anonymised raw data is available.
